# Delayed traumatic rupture of ovarian endometrioma on re-exploration after blunt abdominal trauma

**DOI:** 10.1016/j.tcr.2021.100578

**Published:** 2021-12-08

**Authors:** Vincent Athas, Karen Burtt, John Berne, Jose Lozada

**Affiliations:** Department of Surgery, Broward General Medical Center, 1600 S Andrews Ave, Fort Lauderdale, FL 33316, United States of America

**Keywords:** Open abdomen, Blunt abdominal trauma, Ovarian rupture, Damage control surgery

## Abstract

Traumatic rupture of an ovarian endometrioma is extremely rare injury. We describe a case of a 63-year-old female presenting after a motor vehicle crash (MVC) with complex abdominal injuries requiring exploratory laparotomy that was complicated by delayed presentation of an ovarian endometrioma rupture on second look laparotomy. During the repeat exploration of the abdomen, multiple regions of small bowel and the pelvic floor were noted to be covered with a brown-colored material which was concerning for fecal matter from a missed enterotomy. The patient was kept open for an additional 24 h providing time for occult injuries to reveal themselves and for proper mechanical preparation of the rectum to perform rigid sigmoidoscopy, essential to definitively rule out a missed injury in this rare situation.

## Introduction

Damage control surgery has been an established concept in management of traumatic injuries to the abdomen and is utilized as a tool to break the lethal triad of coagulopathy, acidosis, and hypothermia to which trauma patients are particularly susceptible in the initial stages after injury. This practice has become standard of care internationally in the management of trauma. World Society of Emergency Surgery (WSES) guidelines include persistent hypotension, acidosis, risk for compartment syndrome, or inability to definitively control contamination or necessity of reevaluating bowel viability as indications for damage control surgery in trauma patients [1]. However, as damage control surgery has become more widely utilized, common practice has seen an evolution toward second look laparotomy for occult injury. Here we present an intriguing case of delayed presentation of a ruptured ovarian endometrioma during second look laparotomy which raised concerns for a missed traumatic enterotomy. While traumatic ovarian rupture has been previously described in the literature, it is rare and to our knowledge this is the first time it has been observed in the context of second look laparotomy.

## Presentation of case

**Fig. 1:**
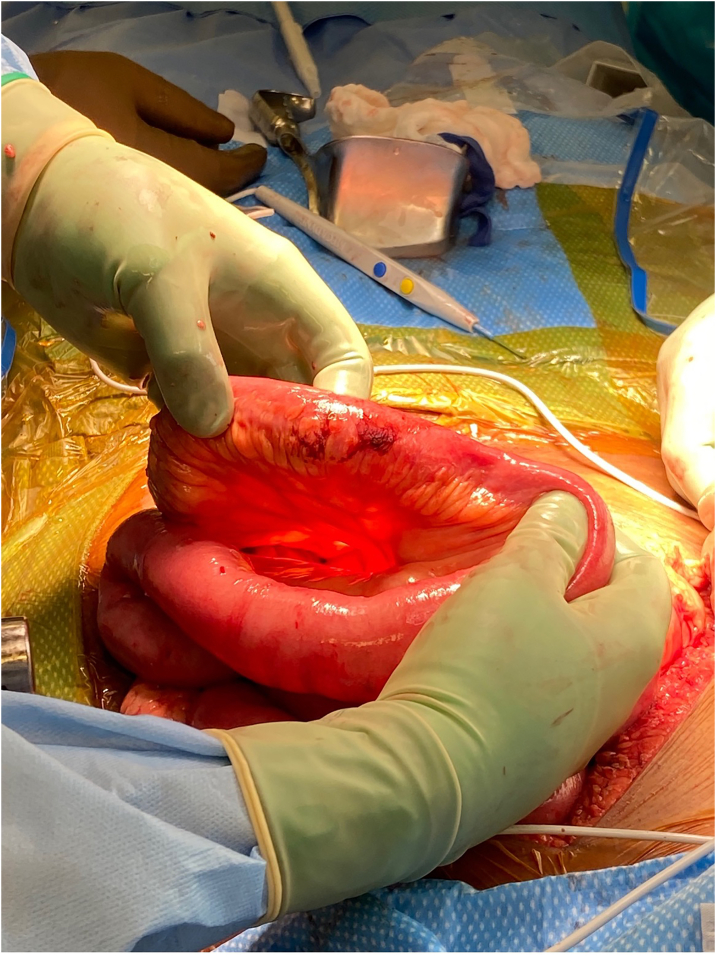
Example of dark staining noted on several regions of small bowel. Examination of the pelvis revealed a cyst like structure adjoining the right ovary oozing dark blood which was excised. The patient was temporarily closed with ABTHERA wound vac for re-exploration 24 h later after mechanical prep of the rectum. We returned to the OR for repeat systematic examination of the abdomen for missed injury and insufflation of the rectum submerged in saline to rule out low rectal enterotomy.

A 63-year-old female retrained passenger was air-flighted from the highway to our level 1 trauma center after a motor vehicle collision (MVC). Patient was a restrained passenger with abdominal distention on initial physical exam but no obvious seat belt sign. Chest x-ray demonstrated bowel in the left chest field and a FAST examination demonstrated free fluid in the pelvis. It was initially difficult to obtain a blood pressure but the patient was noted to have bounding pedal pulses bilaterally with a heart rate of 86, and an oxygen saturation of 99% on 2 l nasal cannula oxygen and the patient was immediately transferred to the operating room for an emergent laparotomy. Examination of the left upper quadrant revealed traumatic rupture spanning the entire left hemi-diaphragm in the coronal plane and a mid-jejunal perforation with minimal contamination. The jejunal perforation was primary repaired and left hemi-diaphragm injury was closed with interrupted #1 Ethibond. It was noted that the patient had a previous gastric bypass with mesenteric tear near the jejunojejunostomy, but the anastomosis appeared viable. A left chest tube was placed and an ABTHERA was placed with the fascia open with the intention to perform a second look laparotomy the next day for to evaluate the jejunostomy anastomosis for viability. Approximately 24 h later the patient was taken back for abdominal re-exploration and at initial examination was found to have dark brown material similar in texture and consistency to feces staining the pelvis and the large and small intestine (Fig. 1). A thorough examination of the abdomen demonstrated no missed injury. A cystic like structure was noted in the right ovary with staining of a similar color and consistency leading to the diagnosis of a ruptured ovarian endometrioma which was excised. However, as it was difficult to differentiate these findings from a missed injury the decision was made to leave the patient open for an additional exploration during which time the rectum could be prepped and the patient positioned for thorough rectal exam and rigid sigmoidoscopy.

The patient remained in the ICU for 24 h and returned to the OR for a 3rd re-exploration in lithotomy position. Again, systematic examination was performed and there was no further staining of the pelvis. Rigid sigmoidoscopy was performed with insufflation of the rectum while submerged in saline. No bubbles were noted. There was no sign of injury and the patient's abdomen was closed. Her postoperative course was uncomplicated. She was discharged on hospital day 28 to an inpatient rehabilitation facility. Final pathology demonstrated normal right sided ovary and an endometriotic cyst.

## Discussion

Second-look laparotomy after complex abdominal trauma allows discovery of delayed presenting devascularization injuries and missed enterotomies. The disadvantages, however, of temporary abdominal closure are numerous including the potential for loss of domain or development of an entero-atmospheric fistula, though more often seen in non-trauma patients [2]. Furthermore, abdominal closure after 5 days has been shown to be associated with 4 times higher anastomotic leak rates [3]. The feculent appearing material during second-look was unexpected but keeping the fascia open for 24 h to allow for repeat exploration we believe was key in safely ruling out missed injury.

Traumatic ovarian cyst rupture is an extremely rare occurrence and is usually associated with an ovarian dermoid cyst or teratoma [4]. In one case there was a report of a 18 female involved in a rollover MVC who was found to have a ruptured ovarian dermoid cyst [5]. In another report a 17-year-old female patient presented after a MVC with mild epigastric pain and was febrile with CT scan findings of a cystic teratoma in the right ovary. She was discharged after 4 days of IV antibiotics with amelioration of her pain, fever, and white count but returned 10 days later with worsening pain and fever. Diagnostic laparoscopy revealed a ruptured ovarian teratoma that was successfully excised [6]. This rare pathology occurs most frequently in younger women, however there is a reported case of a 45-year-old female patient who presented with peritonitis 8 h after a MVC and was found to have a ruptured ovarian teratoma that was successfully excised laparoscopically [7]. Our patient was a 63-year-old demonstrating this rare pathology; post-menopausal women are not immune to this situation. Interestingly, case reports have described a connection between endometriosis and non-traumatic spontaneous diaphragmatic rupture and possibly our patient had endometrial implants on the diaphragm which could have predisposed her to diaphragmatic rupture during the traumatic injury [8]..

Ruptured ovarian cysts can cause significant bleeding. Even ovarian artery ligation to control more significant bleeding is sometimes necessary [4]. Delayed presentation of ovarian artery pseudoaneurysm after MVC treated with coil embolization has also been described [9]. In our case the patient did require transfusion with 2 units of packed red blood cells on post-operative day one after the third laparotomy (fascial closure) for a hemoglobin of 6.7 g/dl. There was significant oozing from the right ovary noted during the second and third laparotomies demonstrating the importance of meticulous hemostasis in these presentations.

This is the first case to our knowledge in the literature that describes discovery of a ruptured ovarian endometrial cyst during second look laparotomy. We propose that, especially in the acute setting of trauma when the risk of missed injury is particularly high, thorough examination of the abdomen during discovery of a ruptured endometrial (“chocolate”) cyst is not enough to rule out missed traumatic enterotomy. Proper rigid sigmoidoscopy in a prepped rectum after an additional 24 h with temporary abdominal closure we believe is essential to ruling out traumatic enterotomy in this context.

## Conclusions

Second-look laparotomy is a central component of trauma surgery management of acute abdominal injury. Traumatic ovarian rupture is extremely rare but can be associated with ovarian cysts. Presentation of a ruptured endometrioma of the ovary is extremely alarming in the context of abdominal trauma but a meticulous and measured approach by keeping the patient in the ICU with temporary abdominal closure, preparing the rectum with plans for re-exploration with rigid sigmoidoscopy and bubble test while insufflating the rectum are central to successfully treating these complex patients and achieving good outcomes.

## Declaration of competing interest

Nothing to disclose.
